# Lipidomes in Cadaveric Decomposition and Determination of the Postmortem Interval: A Systematic Review

**DOI:** 10.3390/ijms25020984

**Published:** 2024-01-12

**Authors:** Leticia Caballero-Moreno, Aurelio Luna, Isabel Legaz

**Affiliations:** Department of Legal and Forensic Medicine, Biomedical Research Institute of Murcia (IMIB), Regional Campus of International Excellence “Campus Mare Nostrum”, Faculty of Medicine, University of Murcia (UMU), El Palmar, 30120 Murcia, Spain; leticia.caballerom@um.es (L.C.-M.); aurluna@um.es (A.L.)

**Keywords:** lipids, fatty acids, long-term PMI, glycerophospholipids, burial sites

## Abstract

Lipids are a large group of natural compounds, together with proteins and carbohydrates, and are essential for various processes in the body. After death, the organism’s tissues undergo a series of reactions that generate changes in some molecules, including lipids. This means that determining the lipid change profile can be beneficial in estimating the postmortem interval (PMI). These changes can also help determine burial sites and advance the localization of graves. The aim was to explore and analyze the decomposition process of corpses, focusing on the transformation of lipids, especially triglycerides (TGs) and fatty acids (FAs), and the possible application of these compounds as markers to estimate PMI and detect burial sites. A systematic review of 24 scientific articles from the last 23 years (2000–2023) was conducted. The results show that membrane glycerophospholipids (such as phosphatidylcholine and phosphatidylglycerol, among others) are the most studied, and the most promising results are obtained, with decreasing patterns as PMI varies. Fatty acids (FAs) are also identified as potential biomarkers owing to the variations in their postmortem concentration. An increase in saturated fatty acids (SFAs), such as stearic acid and palmitic acid, and a decrease in unsaturated fatty acids (UFAs), such as oleic acid and linoleic acid, were observed. The importance of intrinsic and extrinsic factors in decomposition is also observed. Finally, as for the burial sites, the presence of fatty acids and some sterols in burial areas of animal and human remains can be verified. In conclusion, glycerophospholipids and fatty acids are good markers for estimating PMI. It has been observed that there are still no equations for estimating the PMI that can be applied to forensic practice, as intrinsic and extrinsic factors are seen to play a vital role in the decomposition process. As for determining burial sites, the importance of soil and textile samples has been demonstrated, showing a direct relationship between saturated fatty acids, hydroxy fatty acids, and some sterols with decomposing remains.

## 1. Introduction

Lipids play a critical role in biology since they act mainly as a source of energy reserve, and they are part of some structural elements, being the main constituent of most membranes, such as in the case of the nuclear membrane and the membrane of some organelles. They have other essential functions, such as participation in specific processes including cell signaling, material transport, cell proliferation, cell differentiation, metabolism, and thermoregulation [1,2,3]. Although lipids are essential for carrying out various processes in the body, they could also cause certain diseases when deregulation occurs [4,5,6,7,8,9]. 

Lipids are a highly complex group classified according to the International Lipid Classification and Nomenclature Committee (ILCNC) [10], constituting an easy-to-understand lipid classification method. This classification was internationally accepted and adopted [11]. According to this system, eight categories of lipids are found: fatty acids (FAs), glycerophospholipids, glycerolipids, polyketides, sphingolipids, prenol lipids, sterols, and saccharolipids [3,12].

After death, biochemical changes occur inside organisms, such as enzymatic and microbial decomposition, giving rise to the macroscopic changes observed in the corpse. In decomposition, macromolecules (proteins, carbohydrates, and lipids) are degraded into their structural elements, such as phosphates and sugars, glucose, amino acids, and FAs [13,14]. The process of human cadaveric decomposition begins around 4 min after death occurs and can last up to months or years to reach complete skeletonization of the corpse [15], although, in the case of burials, the decomposition process can last between 15 and 25 years. This process is very complex and is influenced by various extrinsic and intrinsic factors, such as the environment in which the corpse is found. Furthermore, the environment can give rise to cadaver conservation processes such as mummification or adipocere formation [16].

Due to the rapid expansion of knowledge and the constant evolution in the study of lipids in forensic sciences, it is essential to conduct a systematic review that allows the available evidence to be synthesized and organized systematically. This provides a comprehensive view of the relevant literature, allowing researchers and professionals to contextualize their work within the current landscape. It offers a solid foundation for future research, identifies emerging trends and gaps in knowledge, and facilitates informed decision-making in forensic practice.

The aim was to systematically review the possible applications of the study of lipidome and its different applications in forensic sciences, to understand the lipid variation profile and the influence of intrinsic and extrinsic factors during the human and animal decomposition process, to conclude the existence of algorithms for the determination of the postmortem interval (PMI) based on the lipid composition, and to determine the existence of a typical profile of lipids and fatty acids (FAs) for establishing burial sites.

## 2. Materials and Methods

### 2.1. Systematic Review

A literature search covering the last 23 years (December 2000 and February 2023) was conducted, following the Preferred Reporting Items for Systematic Reviews and Meta-Analyses (PRISMA) guidelines [17].

### 2.2. Inclusion Criteria

Studies that performed lipidomic and metabolomic analyses were included to determine the lipid and FA degradation profile in both humans and decomposing animals. The criteria were selected based on three main inclusion criteria: (i) studies in postmortem samples with lipid variation profiles during decomposition; (ii) studies on postmortem samples about the determination of burial sites for both humans and animals; and (iii) studies that included, as study matrices, animal and human samples, forensic soil samples, and textiles. Finally, a total of 24 original articles were analyzed.

### 2.3. Search Strategy

The bibliographic search strategy involved a search in the most consulted online scientific databases, PubMed and Google Scholar, a free-access web search engine, and a search strategy developed using keywords.

First, a review of the title, first author, year, and abstract was conducted, considering the inclusion and exclusion criteria. Systematic literature searches were conducted using a combination of keywords, including (“lipidomics” and “PMI”), (“lipidomics” and “postmortem Interval”), (“biomarkers” and “PMI”), (“bone” and “PMI”), (“metabolomics” and “PMI”), (“fatty acids” and “forensic science”), (“fatty acids” and “decomposition” and “forensic”), (“fatty acids” and “decomposition” and “pig”), (“fatty acids” and “cadaveric decomposition”), (“adipocere” and “soil samples”), (“adipocere” and “forensic science” and “fatty acids”), (“adipocere” and “cadaveric decomposition”), and (“cadaveric decomposition” and “soil”).

### 2.4. Data Extraction

Duplicate data were recovered using Microsoft Excel for Office 365. To systematize the most relevant information from each of the studies analyzed, the following data from all the studies that met the criteria were extracted and collected in a table: authors, year of publication, category of the analyzed molecule, type of lipid, sample analyzed (human or animal, soil, and textile fabric), data of the samples taken (sex, PMI, condition of the samples, body mass index (BMI), age, and cause of death), days on which the postmortem sample is taken, main results, and object of study.

### 2.5. Risk of Bias Assessment

The likelihood of bias was assessed by comparing the Cohort Research Checklist Critical Assessment Skills Program (CASP) [18] in each study. The CASP checklist evaluated the following confounding variables: sex, age, environment in which the samples are located, PMI, and cause of death. Study performance was rated as “poor”, “moderate”, or “good” based on the CASP checklist. The overall level of the test was rated as “strong”, “moderate”, “poor”, or “extremely low” [19].

### 2.6. Exclusion Criteria

Studies were excluded based on the following exclusion principles: (a) studies with nonspecific results or inconclusive results (n = 12); (b) studies where the objective of the study was other molecules, such as proteins or amino acids (n = 26); and (c) literature reviews (n = 5). A total of 43 studies were excluded from this systematic review.

### 2.7. Characteristics of the Studies Included in the Systematic Review

**Figure 1:**
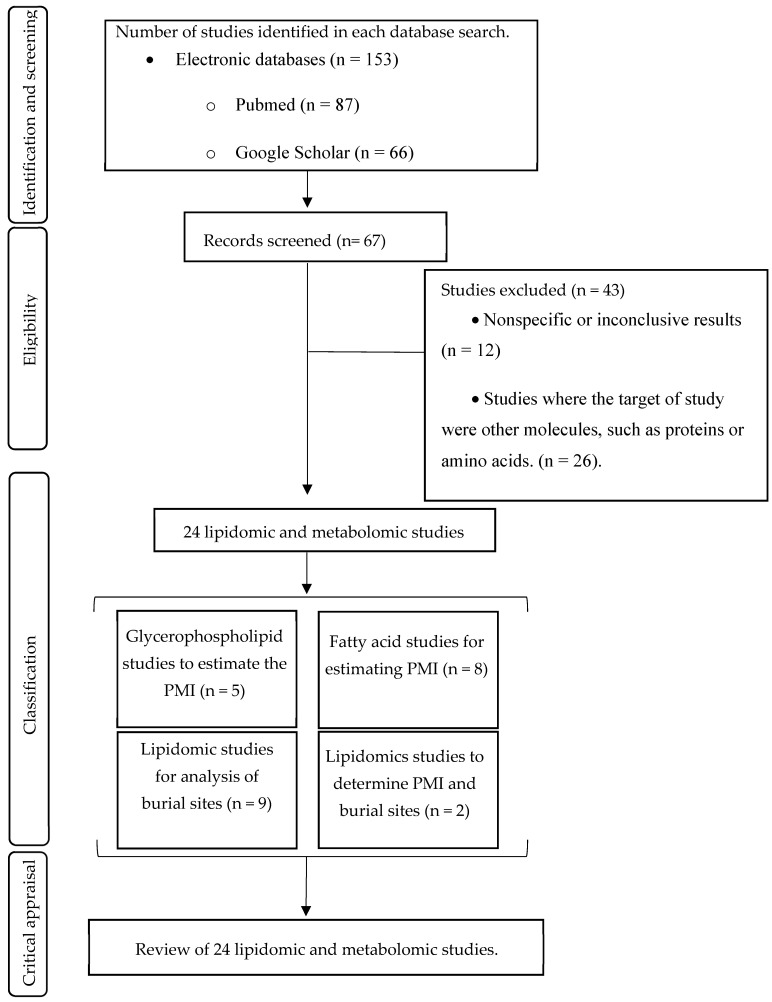
Flow chart of the literature search process and study selection according to PRISMA guidelines.

As shown in Figure 1, 153 studies were identified in two electronic scientific databases, PubMed (87) and Google Scholar (66), and 86 duplicates or nonrelevant studies were eliminated. The titles and abstracts of the remaining 67 studies were reviewed to assess their relevance to the exclusion criteria. When the title did not provide sufficient information, the abstract was revised, or, if necessary, the entire article was examined. Finally, this search strategy identified 24 descriptive studies for inclusion in this systematic review: 5 studies of glycerophospholipids (GPL) about PMI, 8 studies of FA about PMI, 9 studies about sites of burial, and 2 studies estimating PMI and burial sites.

### 2.8. Risk of Bias Assessment

According to the CASP assessment of the probability of bias, most studies (87.5%) were considered “good” or “moderate” based on the variables considered, while 12.5% were considered “poor”, mainly due to confounding variables that were not considered (Table 1). In general, the bibliography exhibited a “good” quality. 

### 2.9. Laboratory Methods

Different biological matrices used for lipid studies were collected (Table 2). The following matrices were gathered: (i) skeletal muscle tissue (4/24), (ii) adipose tissue (1/24), (iii) bone (2/24), (iv) soil from burial sites (13/24), (v) blood (1/24), (vi) textiles in contact with decomposing remains (2/24), and (vii) soft tissues (1/24). The methods used to evaluate the degradation of lipids and FAs in the different study matrices are the following (Table 2): shotgun lipidomic analysis (1/24), GC–MS (gas chromatography/mass spectrometry) (9/24), GC–MS/MS (gas chromatography with triple quadrupole mass spectrometry) (3/24), HR-MS (high-resolution mass spectrometry) (2/24), LC–MS (liquid chromatography/mass spectrometry) (3/24), GC-FID (gas chromatography with flame ionizer detector) (3/24), DRIFTS (diffuse reflectance mode infrared spectroscopy) (1/24), FTIR-ATR (Fourier transform spectrophotometer attenuated total reflectance) (2/24), ICP-OES/ICP-MS (inductively coupled plasma optical emission spectrophotometry/inductively coupled plasma mass spectrometry) (1/24), and FTIR (Fourier transform spectrophotometer) (1/24).

## 3. Results

### 3.1. Corpse Decomposition Process

**Figure 2:**
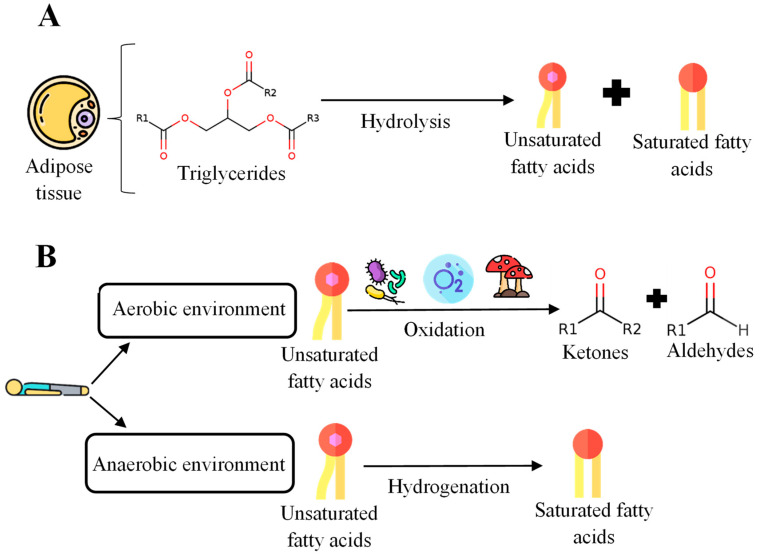
General scheme for the degradation process of postmortem adipose tissue. (**A**) Initial stages of the degradation process of adipose tissue. (**B**) Processes in aerobic and anaerobic environments after death.

Triglycerides (TGs) are formed through glycerol molecules and three FA molecules [15] in adipose tissue that comprise 90–99% of the total composition. After death, hydrolysis reactions occur, leading toa decrease in the concentration of lipids, especially TGs, and an increase in FAs as the decomposition process progresses [42]. The most abundant FA in adipose tissue of the human body is oleic acid (C18:1), followed by linoleic acid (C18:2), palmitoleic acid (C16:1), and palmitic acid (C16:0). This results in variable concentrations of lipids and, therefore, FA, within the body’s cells during cadaveric decomposition [42]. The adipose tissue degradation process is shown below (Figure 2A). After this hydrolysis, two different processes can occur depending on the type of environment (Figure 2B).

In an aerobic environment, unsaturated fatty acids (UFAs) can be oxidized by bacteria, fungi, or atmospheric oxygen. The final products obtained in this reaction are ketones and aldehydes [42]. On the other hand, in an anaerobic environment, the mixture of saturated fatty acids (SFAs) and UFAs generated postmortem can undergo additional hydrolysis and hydrogenation. This hydrogenation process introduces a hydrogen bond that causes unsaturated bonds to become saturated.

**Figure 3:**
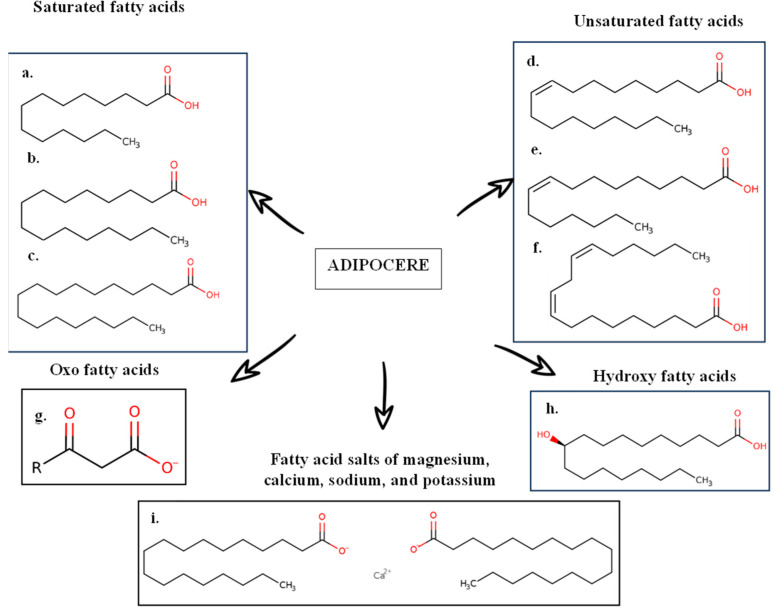
Composition of adipocere. (**a**) Myristic acid; (**b**) palmitic acid; (**c**) stearic acid; (**d**) oleic acid; (**e**) palmitoleic acid; (**f**) linoleic acid; (**g**) 3-oxo-fatty acid anion; (**h**) 10-hydroxystearic acid; (**i**) calcium stearate. Described by [44].

Additionally, when cadaveric decomposition occurs, adipocere formation will occur if the body is in the appropriate temperature, humidity, and soil conditions. Subsequently, it has been proven that body humidity is sufficient for adipocere and can form in almost any environment [39]. Adipocere is a compound that has an essential use in forensic sciences since its appearance makes the corpse’s decomposition slower or, in some cases, allows the preservation of the remains [43]. Adipocire [39] is formed from the degradation of the body’s adipose tissue, and its composition is a mixture of SFAs and UFAs from the degradation of adipose tissue, fatty oxoacids, hydroxy fatty acids, and fatty acid salts. The composition of the adipocere is shown below [44] (Figure 3).

When a corpse is in contact with the ground, compounds generated during decomposition, including lipids, FA, and adipocere, could pass into it via leaching [45]. The soil easily adheres to clothing, the person, or can even remain on certain surfaces or materials, such as vehicles [15,45], making it an accessible sample. It should be noted that lipids and FAs are very stable and can remain for years in the corpse’s remains and its surroundings. Lipids and FAs have been detected in tissue samples taken from mummies [14]. Studies were also found where FAs can be found in the remains of a child from the late Roman period, such as myristic acid (C14:0), palmitic acid (C16:0), and stearic (C18:0) [15,46]. Therefore, due to the degradation process that postmortem lipids undergo and their good preservation over time, they are potential biomarkers for determining the PMI and burial places.

### 3.2. PMI

The PMI is the time elapsed between death and the moment the body is found [13], and its determination is one of the great limitations in Forensic Sciences. Specific mechanisms are used to determine the PMI, the benefits of which vary depending on the stage of decomposition. Determining the short-term PMI has been the subject of some research studies [47]. This has led to the development of some methods for its determination, for example, biochemical studies, such as the analysis of amino acids and tissue organic acids or the determination of potassium in the aqueous humor of the eye, where it is possible to identify a relationship between the potassium concentration in the vitreous humor and PMI. Potassium concentration increases in the vitreous humor after death [47]. However, this method can only be used before the putrefaction process begins [27,47]. After the decomposition stage, the body undergo morphological changes. Some of these changes are the cooling of the body (algor mortis), increased rigidity (rigor mortis), the settling of blood in the sloping areas of the body (livor mortis), and the appearance of a purplish-pink color.

While short-term PMI determination has been widely studied, long-term PMI determination has not proven very interesting [35]. The significant problem in carrying out the determination of the PMI in the long term lies mainly in the inability to observe the morphological changes that are generated once death has occurred, especially if there is a loss of soft tissues of the individual due to scavengers or if the body has been affected by other environmental factors, such as fire, chemical agents, or affected by high temperatures [27].

Recently, there has been an increasing interest in the study of lipids for estimating long-term PMI since lipids undergo a degradation process as cadaveric decomposition occurs. This implies the possibility of finding a pattern of constant change in these compounds that allows us to relate it to the PMI.

### 3.3. Lipid Markers for PMI Estimation

Since tissue lipids are degraded during the breakdown process, leading to FA, and considering the most commonly studied lipids, this section will be divided into studies based on GPL and FA variation profiles.

#### 3.3.1. GPL as Markers for PMI Estimation

Five studies were found that estimated the PMI by studying GPLs. The main results that concern this part of the review are collected in Table 3. The studies are presented in different study matrices and various organisms, encompassing both animals and humans, and involve different experimental conditions: sample treatment, decomposition time, different ages, and sex.

It should be noted that the study times of human decomposition considered in the articles are highly variable, so the articles on human bone and those that analyze skeletal tissue samples were separated.

#### 3.3.2. Human Bone Samples

On the other hand, Table 3 shows studies based on human bone samples. When the degradation process is too advanced, obtaining tissue samples from the corpse is impossible since it can be found in a skeletonized state. Bone is one of the matrices studied with the most interest to determine the PMI.

A recent study [21] analyzed fresh samples of human bone that were left deposited outside for 24 months and determined the presence of GPLs, in this case, PC. It was concluded that degradation of these PCs occurs within the first six months, but it indicates that the critical degradation period occurs between the first three months postmortem, and they are one of the GPLs that generate one of the signals more robust. Furthermore, these compounds can be maintained in bone for decades at low concentrations [21]. This means that PC can be a promising biomarker for determining long-term PMI. Nevertheless, although a clear relationship is observed between the degradation of PC and the PMI, the available data are insufficient to formulate regression equations for predicting the PMI. Therefore, the authors emphasize the need to continue investigating to enable the prediction of PMIs longer than three months [21].

Subsequently, Bonicelli et al. [13] obtained fresh human samples (3–10 days postmortem) from the middle anterior tibia of donors between 61 and 91 years old and deposited them in buried pits or pits open to the outside for 219, 790 and 872 days. This study focused on analyzing the concentration of GPLs (LysoPC, phosphatidylinositol (PI), and PC) in bone. After the analysis, they observed how these compounds’ concentrations decreased [13]. Since this study did not carry out continuous sampling over time, the exact moment of the decrease in the concentration of GPL could not be determined [13]. Differences between samples buried in pits and those deposited outside could also not be determined.

**Figure 4:**
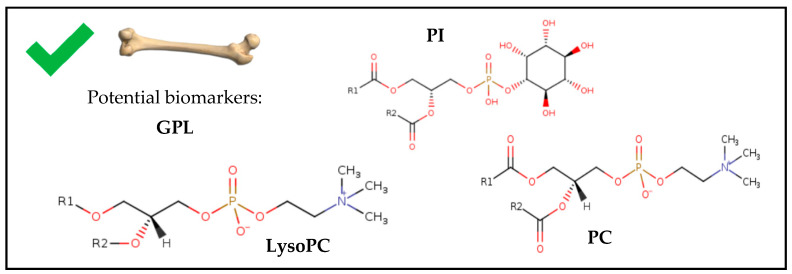
Chemical structures of potential biomarkers for estimating PMI in forensic human bone samples. Chemical structures obtained from ChEBI (Chemical Entities of Biological Interest) [48]. GPL, glycerophospholipid; LysoPC, Lysophosphatidylcholine; PC, phosphatidylcholine; PI, phosphatidylinositol.

Therefore, only two studies have been conducted on human bone samples in which GPL has been analyzed for PMI. This indicates that these compounds decrease as the decomposition progresses (Figure 4), and both studies [13,21] highlight the great potential of GPL as biomarkers for estimating PMI due to their characteristic variation profile with decomposition and their ability to last over time in this sample type.

**Figure 5:**
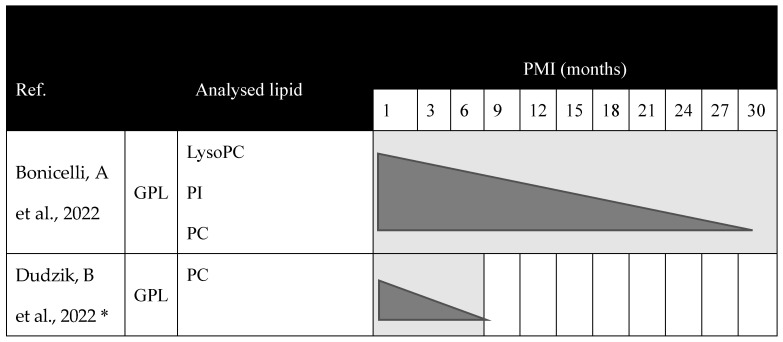
Chronogram of the variation of lipids collected in the systematic review during the process of cadaveric decomposition in human bone samples. * In this case, Dudzik, B et al., 2022 [21] indicates that the critical period for degradation of PC occurs from 0 to 3 months, but degradation occurs during the first six months. References [13,21] are cited in this figure. GPL, glycerophospholipid; LysoPC, Lysophosphatidylcholine; PC, phosphatidylcholine; PI, phosphatidylinositol; PMI, postmortem interval.

Additionally, a timeline shows the variation profile of lipids in human bone samples (Figure 5).

#### 3.3.3. Samples of Human and Animal Muscle Tissue

A study [26] regarding muscle tissue samples (Table 3) located biomarkers for determining PMI using animal (rat) and human skeletal muscle samples in postmortem periods between 3 and 19 days. In this case, the sample variability was more significant, taking samples from men and women of different ages (although with a reasonably narrow interval of 10 years, between 59 and 69 years), with various causes of death and environments of different decomposition.

This study [26] detected GPL as metabolites of interest, including choline phosphate. It observed a pattern of increase in GPL between days 7 and 19 postmortem. This indicated that the muscle tissue used in this study has a high potential for this type of research since it is much more stable than other organs or body fluids [26]. These findings agree with another study [35] where muscle tissue is determined as one of the last to degrade once the organism’s death occurs. It should be noted that, although this study used both human and animal samples in this case, it did not detect any different variation profile between the species; in both cases, choline phosphate increased with decomposition time. They were also unable to detect differences in the pattern of choline phosphate depending on the cause of death or the environment in which decomposition occurs (re-refrigeration or exposure outside).

Another study [27] conducted the first statistical studies of linear regression curves. In this case, human samples of skeletal tissue were also used, which were allowed to decompose until reaching a maximum of 2000 accumulated degree days (ADD) or until enough tissue was left to obtain the sample. Changes in the profiles of some structural GPLs (PC, choline plasmalogen (PlsCh), phosphatidylethanolamine (PtdE), ethanolamine plasmalogen (PlsEtn), and phosphatidylglycerol (PG)) were reported, observing a decrease in these as the decomposition of the corpse progressed. On the other hand, PG and PtdE showed the most consistent signals. Therefore, linear regressions were performed for each of the compounds. The authors indicate that the most useful or reliable linear regression model obtained for determining PMI is with PtdE, where more precise predictions were achieved. This study also carried out combined statistical studies (PG and PtdE). However, according to the results, the combined linear regression curves are not recommended for forensic use since they could not accurately predict the ADD of the validation samples, probably due to multicollinearity. Once again, the suitability of the determination of membrane GPL to calculate the long-term PMI is confirmed, and a linear regression study is obtained for the first time with auspicious results, although still insufficient for use in forensic practice.

Finally, in another study [35], analyzing skeletal muscle samples for 24 days postmortem, it was possible to observe how GPLs, specifically PlsEtn, PldCh, and PG, began to decrease as postmortem time progressed. The authors pointed out other lipid changes, such as the decrease with increasing PMI of cholesterol sulfate, dehydroepiandrosterone sulfate (DHEA), and very long chain saturated fatty acids (VLCFAs), such as hexacosanoic acid and octacosanoic acid. Additionally, there was an increase in UFAs, such as arachidonic acid and cyadonic acid, with the PMI, contrary to what was reported in the previous study [27], where VLCFAs were also detected, but in this case, they began to increase with the PMI, and UFAs began to decrease.

All these discrepancies mean that sterol sulfates are discarded as potential biomarkers by some studies [23,27] due to their greater instability and the complexity of their identification compared to GPL. The same applies to VLCFAs, whose activity is modified by low temperatures [27]. This means that their ability to act as biomarkers is compromised since cadaveric decomposition is affected by changes in temperature [27].

**Figure 6:**
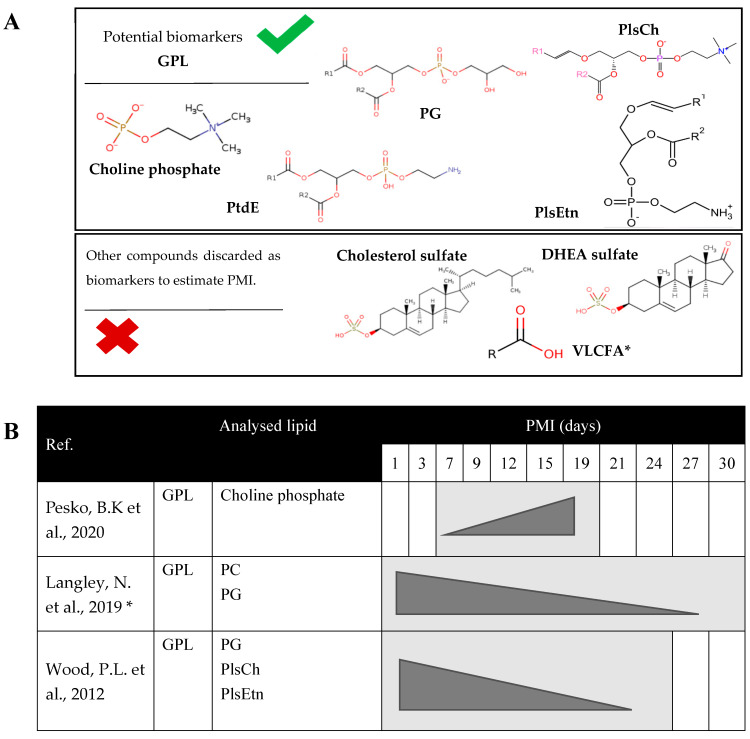
(**A**) Chronogram of the variation of lipids collected in the literature review during the process of cadaveric decomposition in human skeletal tissue samples. * In this case, Langley, N. et al., 2019 [27] does not determine the time (days) of the study is performed but indicates until 2000 ADD or until no tissue is left to sample; specific days cannot be determined until the marker decreases. (**B**) Chemical structures of potential biomarkers for estimating PMI in forensic muscle tissue samples. Chemical structures obtained from ChEBI [48]. The structure of ethanolamine plasmalogen extracted from [49]. DHEA sulfate, dehydroepiandrosterone sulfate; References [26,27,35] are cited in this figure. GPL: glycerophospholipid; PC, phosphatidylcholine; PG, phosphatidylglycerol; PlsCh, choline plasmalogen; PlsEtn, ethanolamine plasmalogen; PtdE, phosphatidylethanolamine; PMI: postmortem interval; VLCFA, very long-chain fatty acids.

Figure 6A shows a compilation schedule of the analyzed studies, showing the variation pattern of GPL according to the decomposition stage (days). Figure 6B shows the main results regarding the biomarkers that can be used with greater reliability in muscle tissue samples to determine PMI and those discarded by the articles in this review.

After reviewing lipid studies, it could be observed that GPL has a high potential to be a biomarker to estimate PMI in bone and muscle tissue samples [13,21,26,27,35]. Nonetheless, many more studies should be conducted to determine the variation profile as decomposition occurs since some controversies were found in the pattern of variation of these compounds and many variations in the studies carried out to date.

#### 3.3.4. FAs and Sterols as Markers for PMI Estimation

The degradation process of postmortem lipids suggests that studying free FA would also be an excellent approach to finding biomarkers that allow us to carry out PMI data. Eight studies were found that determine changes in FA concentrations during decomposition. The different studies are shown in Table 4.

Likewise, these studies are conducted on animal/human tissues and other samples, such as soils from decomposition sites and textiles in contact with corpses. For its study, this section was divided according to the type of sample analyzed.

#### 3.3.5. Samples of Human and Animal Tissues

A study carried out by Ueland et al. [23] determined the decomposition in the external environment of human tissue samples from different parts of the organism (upper arm, lower abdomen region, and area of the buttocks/upper thigh) (Table 4). In this case, two human donors of different ages (68 and 77 years) and, in both cases, with obesity (BMI > 30 (kg/cm^2^)), were examined. The trial time in this case was 69 days. It showed that the concentration of SFAs (stearic and palmitic acid) was much higher than that of UFAs (oleic and linoleic acid), indicating that as decomposition occurs, hydrolysis of the tissue TGs and hydrogenation of UFAs [23,42]. It concluded that SFAs (stearic and palmitic acid) and UFAs (oleic and linoleic acid) are excellent markers for estimating PMI. It also detected other lipids such as sterols (cholesterol, 5a-cholestanol, and cholestanone); but, due to the lack of specificity in the identification of these compounds in the donors, they ended up concluding that these compounds were not good markers for estimating the PMI [23], agreeing with what was previously stated above.

Ueland et al. [23] obtained statistically significant results between the samples from the fresh and frozen donors, where the concentrations of FA were lower in the frozen donor than in the fresh one. In another study, [24] used animal (rat) adipose tissue samples in a controlled environment. The test period (Table 4) varies between 1 and 14 days for all samples except those at 35 °C. In this case, it was predicted that adipose tissue at 35 °C would degrade in about four days. Therefore, the test time was reduced to 35 °C from 1 to 4 days. It was [24] concluded that with longer PMIs, there is an increase in the peaks associated with free FAs and a decrease in the peaks referring to the bonds between FA and glycerol [24].

Furthermore, it was determined that temperature dramatically influences the determination of the PMI in the tissue. In the high-temperature group (35 °C), adipose tissue changes more over time, while at low temperature (5 °C), it changes less. It can be observed that temperature influences the degradation of adipose tissue, as in the previous study [23].

Wu et al. [30] analyzed blood samples from 84 male and female rats, with a postmortem testing time of up to 72 h. They determined the increase in some compounds, such as glycerol, palmitic, stearic, and oleic acid. The PMI of the prediction group of males and females was estimated with a difference betw4en the estimated and actual PMI of 8.18 and 4.04 h, respectively, and an error in the measurement of males and females of 3.699 and 4.99 h, respectively.

Finally, a study was carried out with samples of pig fatty tissue. In the presence and absence of insects, these remains were left outdoors to decompose. The sampling period reaches a maximum of 111 days, approximately four months. In this case, samples were taken from the upper and lower torso to determine if there were differences in the FA profile. It was possible to detect a decrease in the concentration of UFAs (oleic, palmitoleic, and linoleic acid) with the consequent increase in SFAs (stearic and palmitic acid), agreeing with what was previously stated about the processes of hydrolysis and hydrogenation as a result of the degradation of adipose tissue [14].

Nevertheless, it is worth mentioning that this study was carried out in triplicate, and after applying chemometric methods (PCA), the authors concluded that no characteristic FAs could be associated with specific stages of decomposition. It emphasized that “these results do not deny the potential of these markers to estimate PMI”, only that it is necessary to carry out many more research studies to determine individual or group trends in FA [14].

**Figure 7:**
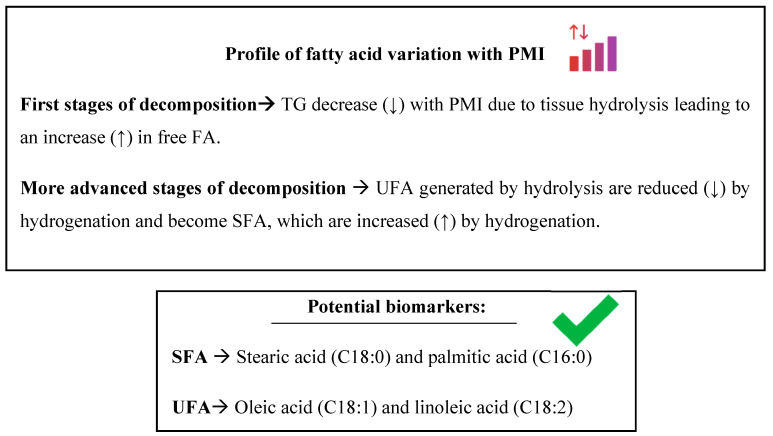
Summary of fatty acid variation profile results in animal and human tissue samples (muscle, blood, and adipose). FA: fatty acid; SFA: saturated fatty acid; TG: triglyceride; UFA: unsaturated fatty acid; (↑): increase; (↓): decrease.

Furthermore, there is a degradation profile of the body’s tissues (Figure 7), where hydrolysis of TG occurs, releasing FA. This generates a mixture of both UFAs and SFAs. Subsequently, UFAs undergo hydrogenation, giving rise to their analogous SFAs. Nevertheless, due to the variety in the testing times of the studies (minimum two and maximum 111 days), it could not relate variations in FA to a specific time (days); we can only talk about early and late stages in the decomposition, meaning that a schedule of the FA variation profile cannot be constructed.

One can also conclude the possibility of obtaining regression models that estimate the PMI with reliability and robustness. However, it should be remembered that some reviewed publications that obtain these promising results carry out metabolic studies, so they do not only consider PA [30].

#### 3.3.6. Other Types of Samples

In other studies (Table 4), the study matrix was soil samples in contact with decomposing remains or textiles in contact with the animal corpse.

Collins et al. [20] determined the variation of FAs from cotton textiles in contact with two corpses, according to the following stages of decomposition: “early”, “medium”, and “late” (classified according to the characteristic morphology observed in the corpses). A profile of reduction in UFAs (palmitoleic and linoleic acid) was obtained with an increase in analogous SFAs (palmitic and oleic acid) from the “early” to the “late” stage. This study also detected increased bile acids (deoxycholic and lithocholic acid), suggesting that “the gastrointestinal tissues were disintegrated and the release of fecal matter was occurred as a part of the decomposition fluids present in the textiles”. In contrast, a completely different profile was observed in winter, showing again that temperature is a very influential factor in the decomposition process.

Other FA detected were volatile fatty acids (VFAs); a study [31] ensures that they are good markers due to their longevity, reproducibility, and environmental stability. VFAs are short-chain fatty acids, between two and five carbons, that can be detected in the middle and late postmortem stages [42]. The soil in contact with a corpse was analyzed in a study conducted by Vass in 2017 [31]. No information about the corpse or the PMI was known in this case. However, some VFAs are present in these samples, specifically propionic acid, isobutyric acid, n-butyric acid, isovaleric acid, and n-valeric acid. This study ensured the possibility of using the analysis of the presence of VFA to estimate the PMI of a corpse if it is used in conjunction with other methodologies, such as visual inspection of the corpse and forensic entomology. According to the study results, these compounds may derive from the decomposition of fat or muscle during cadaveric decomposition or from the microbial decomposition of foods in the intestine, and their presence may be affected by the diet of the individuals.

Through the analysis of soils belonging to human graves exhumed from PMI between 6 and 12 years, [39] concluded that there were differences between soils belonging to lower and higher PMI graves. SFAs (myristic acid, palmitic acid, and stearic acid), UFAs (oleic acid), and 10-hydroxystearic acid were detected in all soil samples. Conversely, the concentration was higher in the tomb samples from 6-year-old PMI and lower in those from 12-year-old PMI. This would indicate that the FA changes in concentration depending on the burial time, producing a decrease over time.

On the other hand, a study [25] used animal samples (Sus scrofa) to simulate burials in vessels with two different types of soil: acidic soil and slightly alkaline, with maximum test times of 28 days. This study demonstrated similar decomposition rates in the two soil types. Furthermore, an increase in the concentration of SFAs (palmitic acid and stearic acid) and monounsaturated fatty acids (MUFAs) (oleic acid) during decomposition was identified. However, the decomposition profile varied between acidic soil and slightly alkaline soil. In acidic soil, the increase in FA occurs in the first stages of decomposition, and in alkaline soil, in later stages, showing that the soil type affects the lipid profile during decomposition.

Another study [29] studied the degradation profile using clothing (textile) that was in contact with the animal corpse (pig) as a study matrix. In this case, three tests were carried out, two in summer and one in winter. The results indicate different decomposition patterns for the two seasons. In summer, an increase in the percentage of SFA was detected in the early stages (between 4 and 31 days) of decomposition, remaining stable until the end of the test, and a decrease in oleic acid. In winter, a different decomposition profile occurs, with an initial decrease in the percentage of SFA and an increase in oleic acid (until day 65). Therefore, it can be determined that temperature is also an essential factor affecting the decomposition process and the variation profile of FAs.

Finally, Ismail et al. [33] studied sandy soil samples in contact with decomposing animal remains (pig) in a test period of 3 months. This study concluded that there were statistically significant differences (*p* < 0.05) in the concentrations of SFAs (stearic acid and palmitic acid) in the different stages of decomposition. Furthermore, a change profile of increase in SFA was detected, reaching the maximum concentration on day 20 of decomposition, subsequently producing a decrease until the end of the trial (day 90).

**Figure 8:**
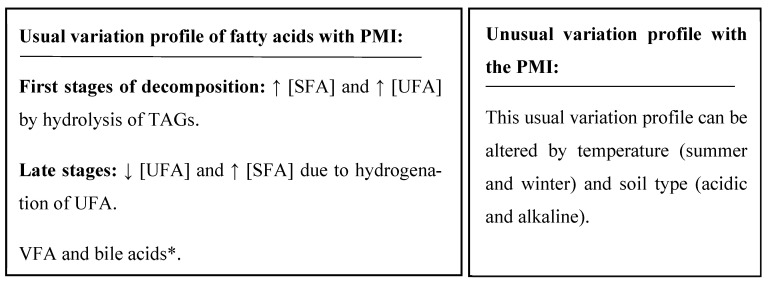
Summary of the results of the variation profile of fatty acids in soil and clothing samples in contact with corpses. * These compounds are not identified in all studies, so there are insufficient references to consider them good markers. [SFA]: concentration of saturated fatty acids; [UFA]: concentration of unsaturated fatty acids; VFA: volatile fatty acid; (↑): increase; (↓): decrease.

The pattern obtained in the soil and textile samples that are in contact with the corpse agrees with the decomposition profile that occurs in human and animal tissues—initially producing an increase in SFA, reaching a maximum, and subsequently producing a decrease over time (Figure 8).

### 3.4. Lipid Markers for Analysis of Burial Sites

Table 5 shows the main studies with the results obtained to determine burial sites or cadaver decomposition islands (CDI).

A soil study [22] was conducted on clandestine graves with an unknown PMI. Samples were obtained from different areas of the grave (in the center, under the feet and head of the corpse, in different directions, partially away from the grave, and at different depths), along with control samples away from where the corpse was located. This study focused sterol analysis and concluded that coprostanol and epicoprostanol were present in the samples from the tombs and those partially distant. Nevertheless, it was not detected in the control samples. This agrees with other review studies reporting that sterols are of interest as markers for identifying burial areas or decomposition fluids [15]. Therefore, sterols can indicate the presence of decomposing matter and affirm that the compounds associated with the decomposition of a body can pass into the soil via leaching [22]. Another study, based on the determination of the concentration of sterols, specifically cholesterol, carried out the burial of a human corpse. Control samples were taken (before depositing the corpse), and soil samples were collected from areas in contact with the corpse. The test time was 14 days. Higher cholesterol concentrations were detected in soil samples from graves than in control samples, indicating that the cholesterol would originate from the body’s decomposition [28]. Another publication detected the relationship of some FAs with the islands of corpse decomposition [32]. This study analyzed control soil samples and samples taken at different depths from the area under the thorax and under the abdomen of a corpse with an estimated PMI of 11–18 days. Samples were taken 18 days postmortem, and final samples were taken at 358 days. The authors reported a higher concentration of FA in the soil samples under the corpse compared to the control samples. In this case, the most abundant FA were acids with 14–18 carbons (C14–C18), mostly SFAs, and 10-hydriostearic acid (Table 5). Oleic acid was also detected, although in a minority, 18 days postmortem. This indicates that cadaveric reduction occurred since UFAs can still undergo hydrogenation to produce SFAs. At 358 days, a greater amount of SFA was detected, indicating a more advanced stage in decomposition, where UFAs had already been hydrogenated. This suggests that FAs from the decomposition process can leach into the soil and persist in the soil for an extended time; in this case, it was detected up to almost one year later. On the other hand, one study detected VFAs only in soil samples from under-decomposing corpses and not in control samples [31].

Algarra et al. [36] aimed to determine the compounds found in CDI soil samples. Soil samples were taken from cemeteries in areas adjacent to tombs and soils containing adipocere. This study found that in this type of soil, many FAs can be identified and, in the case of soils with adipocere, 10-hydroxystearic acid can be detected, already identified in past studies [50] as a component of the adipocere.

A study [39], which has already been mentioned in PMI estimation, detected SFAs (myristic, palmitic, and stearic acid), UFAs (oleic acid), and the presence of 10-hydroxystearic acid in the exhumation soil samples. At the same time, it was absent in the control samples (samples taken from the walls of the tombs).

Forbes et al. [40] analyzed soil samples from exhumations with PMI ranging between 5.5 and 50 years from different environments (wet and dry) and forensic samples with unknown PMI. This study detected the presence of FAs, with the majority being palmitic acid in almost all the samples, while control samples showed a total absence of these compounds. It also highlighted the presence of differences in the concentrations of acids depending on the environment from which the soil sample originated, specifically in humid and dry environments. In humid environments, there was a higher concentration of palmitic acid and a lower concentration of stearic acid compared to dry environments [15,40]. This study also pointed out that a higher concentration of palmitic acid and a lower concentration of stearic acid indicated a later stage in adipocere formation [40]. On the other hand, when oleic acid was more abundant than the rest of the FAs, it was indicative that the adipocere formation process had not yet begun. This is supported by another study [51], which indicates that the degradation of oleic acid must occur to form adipocere.

Regarding the analysis of human samples, a study [41] used soil samples from exhumed graves with PMI of 27 months, 12, 22, and 26 years were used. This study detected the presence of FAs, fatty acid salts, and hydroxy fatty acids. Among the FAs, the most abundant in all the samples analyzed was palmitic acid, but others, such as myristic or stearic acid, were also detected.

On the other hand, studies on animal samples were also reviewed [34]. Soil samples from animal burials of four pigs were analyzed. The test time was 3 months for one group (two bodies) and 6 months for another (two bodies). The presence of sterols (cholesterol and β-sitosterol) was detected in the soil samples in contact with the 3-month PMI bodies with statistically significant differences in concentration (*p* < 0.05) in the control samples. Coprostanol was also detected in some samples, while it was absent in the control sample. In the 6-month PMI samples, the presence of cholesterol and β-sitosterol was detected but in a lower concentration, while coprostanol could not be detected; a variation with the PMI can also be determined. Due to the presence of sterols in the study samples and not in the control samples, the authors [34] assumed that it originated from decomposing corpses.

Stuart et al. [37] conducted a trial where they buried samples of pig adipose tissue in soils, simulating burials in two different environments (silty soil and a coffin burial) for 12 months. This study determined that during the stages of adipocere formation, the concentrations of TGs, FAs, fatty acid salts, oxyacids, and hydroxy fatty acids varied. TG entered mainly in the first stages of adipose formation, with lower TG concentration and higher SFA and UFA levels in the intermediate stages. Finally, in the most advanced stages of formation, the presence of SFAs, fatty acid salts, oxyacids, and hydroxy fatty acids was determined [37].

Another study analyzed soil samples from places of animal decomposition (pig) and samples of adipocere formed directly on the corpse, with different PMIs (5, 6, 8, 13, and 14 months) [38]. this study determined that in the soil samples, remains of the adipocere formed from the decomposing corpse, and variations in FAs can be found during different stages of adipocire formation. In the initial stage, there was a decrease in UFAs (mainly oleic acid) and an increase in SFAs (primarily palmitic acid). In the intermediate stage, there is an increase in stearic acid, a reduction in oleic acid due to the hydrogenation process, and a total decrease in palmitoleic and linoleic acid (UFAs). In the final stage, there was a reduction in the concentration of oleic acid, the total absence of linoleic and palmitoleic acids, and an increase in the concentration of palmitic acid (representing more than half of the total FA composition).

**Figure 9:**
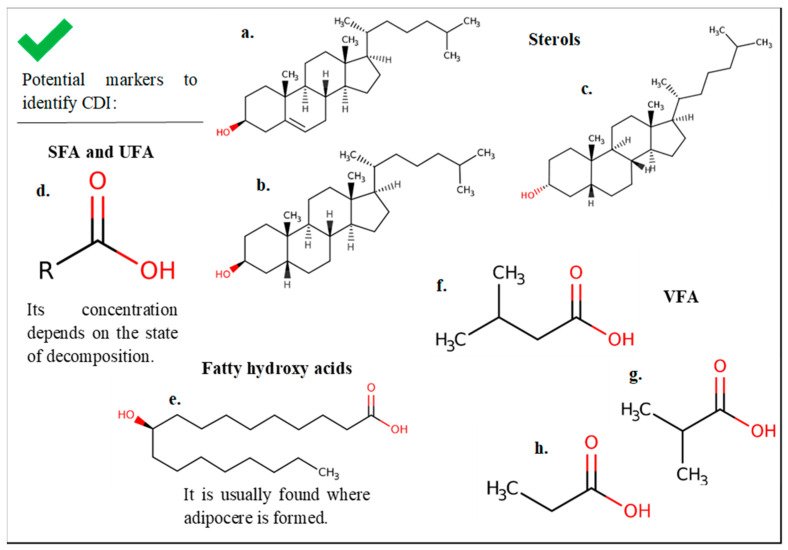
Summary of the main results of soil studies at human and animal burial sites. (**a**) Cholesterol, (**b**) coprostanol, (**c**) epicoprostanol, and (**d**) fatty acids will be unsaturated (double bond) or saturated (single bond) depending on the bonds in the chain represented by R. (**e**) 10-hydroxystearic acid. (**f**) Isovaleric acid. (**g**) Isobutyric acid. (**h**) Propionic acid. Chemical structures obtained from ChEBI [48]. CDI: cadaver decomposition island; SFA: saturated fatty acid; UFA: unsaturated fatty acid; VFA: volatile fatty acid.

In conclusion, FAs produced during decomposition in body tissues leach into the environment. The soil samples studied verified that the presence of FAs, sterols, and other compounds, such as 10-hydroxystearic acid, can be suitable markers for determining places where decomposing remains are found (Figure 9).

## 4. Discussion

The aim of this study was to systematically review the possible applications of lipidome research and its different applications in forensic sciences. The focus was on understanding the lipid variation profile and the influence of intrinsic and extrinsic factors during the human and animal decomposition process. This review also aimed to explore the existence of algorithms for the determination of the postmortem interval (PMI) based on the lipid composition and determine the existence of a typical profile of lipids and fatty acids (FAs) for establishing burial site.

Regarding the estimation of the PMI, numerous articles [13,14,20,21,23,24,25,26,27,29,30,31,33,35,39] classify GPLs and FAs as potential biomarkers. Most studies detect a decrease in GPL with increasing PMI [13,21,27,35]. This is attributed to the activation of some enzymes during necrosis, such as phospholipase A-2 (PLA2), whose primary function is the hydrolysis of phospholipids, specifically those containing an ionic group linked to phosphate, such as PC and PG [52]. However, a contradictory increase in choline phosphate was reported in another publication [26]. According to these authors [26], this compound can be formed in two ways: from choline through a reaction requiring ATP (adenosine triphosphate), which is unlikely in dead tissue due to ATP absence; or from PtdE [26] through GPL metabolism. These two pathways could justify why, in the study by Langley et al. [27], there was a decrease in PtdE, while Pesko et al. [26] detected an increase in choline phosphate.

Regarding FAs, a clear degradation profile is determined within the tissues of the postmortem organism. TG decreases in concentration, producing increased SFAs and UFAs due to hydrolysis. As the decomposition time increases, there is a decrease in UFAz since it is transformed into their analogous SFAs through hydrogenation, which is in agreement with numerous studies [15,42,44]. Then again, SFA and UFA concentrations differ depending on the body’s decomposition state. It is also reflected in soil samples from burial sites where these lipids in the decomposition fluids enter the environment through leaching [15,45] and can remain for a long time [14,15,53]. It makes them potential markers of decomposition fluids.

Nevertheless, the reviewed articles show that the variation profiles between animal (pig) and human samples are very similar. This indicates that the markers are present in both species, which is an advantage due to the similarity in the decomposition process that seems to exist between pigs and humans [54]. This similarity makes these animals suitable for carrying out this type of study due to the existing ethical limitations associated with the use of human bodies. Nonetheless, it could also pose a problem when identifying clandestine human graves from animal burials.

Several studies found that sterols, such as cholesterol, coprostanol, epicoprostanol, and β-sitostanol, are markers of decaying matter [20,22,28,34]. Still, many controversies surrounding sterols were found regarding their use as biomarkers of decomposition fluids. In some studies [55], sterols, including Δ5-sterols, cholesterol, β-sitosterol, and stigmasterol, were detected in soils from World War graves. After the study, the authors [55] concluded that it was part of the “natural sterol background” of that soil, excluding them as decomposition markers. Other studies [34] indicated that β-sitostanol, an element of plant origin, can come from the intestine of animals if they have ingested plant matter in their diet. However, it can also come from the plant matter present in the soil of the place, so they are also discarded as biomarkers due to possible contamination of the environment. In contrast, some studies [20,28] selected cholesterol as a potential marker of decomposition fluids. Some authors [20] attribute these controversies to the age of the samples, with some being of archaeological origin [55] and others more recent [20,28].

Despite the typical results found, it is worth mentioning that the studies analyzed were very different. In GPL studies, varied results were obtained regarding the types of GLP analyzed. Lipidomics, when applied for this objective in forensic sciences, is still in development, which suggests that the studies initially try to detect the most significant amount of GPL and FA to subsequently study their potential as biomarkers for estimating PMI and identifying burial sites.

In the case of GPL for estimating PMI, only two samples were analyzed (human bone and muscle tissue), concluding that it is an excellent tissue to carry out these studies. Future studies should include other types of samples to investigate if the study matrix influences these lipid change profiles. In the case of FA, a more diverse set of samples, including blood, textile, muscle tissue, adipose tissue, and soil samples, was used for estimating PMI and burial sites. Nevertheless, the variation profiles showed a greater consistency while being very similar in all the studies analyzed.

One of the most limiting factors found is the minor variation in the cohorts of samples taken and the few significant results indicating their influence on the lipid variation patterns, and numerous intrinsic and extrinsic factors can affect the decomposition process [14]. Regarding intrinsic factors that may influence, some scientific evidence suggests that the lipidome changes depending on sex and age [56]. While age is a less influential factor in the lipidome, sex is known to be one factor that most affect the lipidomic and metabolomic profiles [57]. Statistically significant differences between men and women have been found in lipids in blood plasma (PC, PtdE, sphingomyelin, and ceramide), with higher concentrations found in women [56]. In studies that include sex differences [26], the authors do not mention any representative changes in the lipidome. Indeed, the number of individuals analyzed was not very representative to observe statistically significant changes since only one female case was studied compared to five males; in addition, the number of total samples analyzed was relatively limited (six samples).

Another study [14] reports statistically significant differences in the concentration of FAs between samples from the upper torso and the lower torso (pig) [14], and Von der Lühe et al. [32] detect differences in the concentration of FA in the soil under the thorax and abdomen, with higher levels under the thorax. Some studies show that the composition of tissues differs due to their function within the organism [58]. Furthermore, others show statistically significant differences between some SFA and UFA concentrations in tissue areas [59]. Finally, studies [60] show that the difference in the thickness of the tissue analyzed can alter the levels of SFA and UFA present, which would agree with the results obtained in the reviews.

Although the reviewed studies do not conclude relevant information about the study variable “cause of death”, there is scientific evidence that indicates that depending on the cause of death, the generated metabolic profiles are different [61], so it would be interesting to assess this factor in future studies. It would also be interesting to study the influence of BMI since a possible relationship with the lipidome has been found. A study [56] concluded that 47 lipids were related to body mass index, including LysoPC, PC, and PtdE [56]. Nonetheless, no relationship with a different lipid breakdown pattern is mentioned in the reviewed articles.

Regarding extrinsic factors, a great influence of temperature on lipid variation profiles was determined [23,24,29]. These studies were based on applying different temperatures, conducting tests in different seasons (summer or winter), and using samples preserved in different states (fresh and frozen). Yu et al. [24] indicate that these differences are mainly due to the increase in the degradation rate of adipose tissue with temperature; since the degradation is faster, the technique can detect the compounds better. This difference in the speed of degradation associated with temperature is also shown in another article [20], where the authors assure that in winter, where temperatures are lower, degradation is delayed, generating a lower concentration of SFAs and UFAs in the samples. In this regard, other studies were found that indicate that in winter, alternative mechanisms can be generated in the degradation of FAs [29], where, instead of hydrogenation, the dehydrogenation of stearic acid occurs by an oxidation process due to microbial enzymes present in the first postmortem stages [29]. In other studies, these alternative mechanisms were established years ago [29,62,63]. Therefore, the influence of temperature is significant and must be controlled in subsequent studies to determine a pattern of lipid variation at different temperatures and seasons. Comstock [14] shows significant differences between decomposition in the absence and presence of insects, concluding that a higher hydrogenation rate occurs when insects are present. Furthermore, some studies indicate that the speed of decomposition is more incredible in the presence of insects [14,64]. According to this study [14], this occurs because insects generate more significant tissue loss or also due to the contribution of microorganisms that can contribute to the degradation of FAs.

Regarding the type of soil, differences were noted in the analyzed studies regarding the concentration of FAs between wet and dry soils [40] and soils with different pH levels [25]. Evidence was found regarding the influence of water on the formation of adipoceras, which can alter the concentration of FAs in humid soils. [40,65,66]. this agrees with Forbes et al. [40], where differences in the concentrations of palmitic and stearic acid were found between humid and dry soil, and in [29], wherein stations with greater precipitation produced a detection of FA later in time, indicating a delay in the decomposition process. Scientific evidence was found [67] about pH, which indicates that in acidic soils, the decomposition rate is up to five times greater than in alkaline soils, contrary to what occurred in [25]. According to the authors, degradation rates were higher in relatively alkaline soils [25] due to the possible influence of other environmental factors. About the soil samples, scientific evidence indicates that the concentration of lipids decreases as the depth of the soil increases [45], agreeing with the results found in a publication of this review [32], where the detected compounds decrease as the depth at which the soil sample increases. It is also essential to know the depth of the sample.

We can conclude the importance of incorporating, in future studies, environmental variables (temperature, seasons, humidity, pH, and depth at which the body is buried) and those of the donors (cause of death, sex of the donors, and age) to determine their influence on the degradation process. This consideration is essential when performing a multi-linear regression model to estimate the PMI.

## 5. Conclusions

In conclusion, lipids, fatty acids, and other compounds are valuable biomarkers to estimate the postmortem interval (PMI) and explore burial sites. The formation of adipocere from the degradation of adipose tissue is highlighted as a possible significant marker for estimating PMI by slowing down the decomposition. The importance of the distinction between aerobic and anaerobic environments during decomposition is observed since they exert an impact on the concentration of lipids and fatty acids. This difference is evident in the oxidation of unsaturated fatty acids (UFAs) in aerobic conditions and additional hydrolysis in anaerobic conditions. On the other hand, the various matrices explored, such as bones, muscle tissue, and soil, have identified lipids and fatty acids as stable biomarkers for determining PMI. The difficulty of estimating long-term PMI is highlighted due to soft tissue loss and the influence of environmental factors. Fatty acid concentration changes during decomposition suggest a distinctive profile where unsaturated fatty acids increase before hydrogenation, increasing saturated fatty acids. Sterols, fatty acids, and other compounds are considered potential indicators in samples of grave soil with different PMIs. Furthermore, although glycerophospholipids and fatty acids are explored as potential markers, continued research is needed to establish reliable regression models. Finally, the significant influence of temperature, environment, and decomposition stage on lipid profiles is highlighted, emphasizing the inherent complexity in establishing universal markers for PMI estimation. 

## Figures and Tables

**Table 1:** Risk of bias assessment.

**Ref.**	**The Study Addresses the Problem.**	**Acceptable Cohort Recruitment**	**Precisely Measured Exposure**	**Precisely Measured Results**	**Important Confounding Factors Identified**	**Critical Confounding Factors Are Taken into Account**	**Accurate Results**	**Credible Results**	**The Results Agree with Other Available Data**	**Overall Quality Score**
[20]	**✓**	**🗴**	**✓**	**✓**	**✓**	**✓**	**✓**	**✓**	**✓**	Good
[13]	**✓**	**🗴**	**✓**	**🗴**	**✓**	**✓**	**✓**	**✓**	**✓**	Good
[21]	**✓**	**🗴**	**🗴**	**✓**	**✓**	**✓**	**✓**	**✓**	**✓**	Good
[22]	**✓**	**🗴**	**🗴**	**🗴**	**🗴**	**🗴**	**✓**	**✓**	**✓**	Moderate
[23]	**✓**	**🗴**	**✓**	**✓**	**🗴**	**🗴**	**✓**	**✓**	**✓**	Good
[24]	**✓**	**🗴**	**✓**	**✓**	**🗴**	**🗴**	**🗴**	**🗴**	**✓**	Moderate
[25]	**✓**	**🗴**	**✓**	**✓**	**🗴**	**🗴**	**✓**	**✓**	**✓**	Good
[26]	**✓**	**✓**	**✓**	**✓**	**✓**	**✓**	**✓**	**✓**	**✓**	Good
[27]	**✓**	**🗴**	**✓**	**✓**	**🗴**	**🗴**	**✓**	**✓**	**🗴**	Moderate
[28]	**✓**	**🗴**	**✓**	**✓**	**🗴**	**🗴**	**✓**	**✓**	**✓**	Good
[29]	**✓**	**🗴**	**✓**	**✓**	**✓**	**✓**	**✓**	**✓**	**✓**	Good
[30]	**✓**	**✓**	**🗴**	**🗴**	**✓**	**✓**	**✓**	**✓**	**✓**	Good
[31]	**✓**	**🗴**	**✓**	**🗴**	**🗴**	**🗴**	**🗴**	**🗴**	**✓**	Poor
[32]	**✓**	**🗴**	**✓**	**✓**	**🗴**	**🗴**	**✓**	**✓**	**✓**	Good
[33]	**✓**	**🗴**	**✓**	**✓**	**🗴**	**🗴**	**✓**	**✓**	**✓**	Good
[14]	**✓**	**🗴**	**✓**	**✓**	**🗴**	**🗴**	**✓**	**✓**	**✓**	Good
[34]	**✓**	**🗴**	**🗴**	**✓**	**🗴**	**🗴**	**✓**	**✓**	**✓**	Good
[35]	**✓**	**🗴**	**🗴**	**🗴**	**🗴**	**🗴**	**🗴**	**🗴**	**🗴**	Poor
[36]	**✓**	**🗴**	**🗴**	**🗴**	**🗴**	**🗴**	**✓**	**✓**	**✓**	Moderate
[37]	**✓**	**🗴**	**🗴**	**🗴**	**🗴**	**🗴**	**🗴**	**🗴**	**✓**	Poor
[38]	**✓**	**🗴**	**🗴**	**✓**	**🗴**	**🗴**	**✓**	**✓**	**✓**	Good
[39]	**✓**	**🗴**	**✓**	**🗴**	**🗴**	**🗴**	**✓**	**✓**	**✓**	Moderate
[40]	**✓**	**🗴**	**✓**	**✓**	**🗴**	**🗴**	**✓**	**✓**	**✓**	Good
[41]	**✓**	**🗴**	**✓**	**🗴**	**🗴**	**🗴**	**✓**	**✓**	**✓**	Moderate

Data based on CASP-based Risk of bias assessment. **✓**, It was possible to evaluate this variable **🗴**; It was not possible to evaluate this variable.

**Table 2:** Description of different biological matrices and analytical techniques used to detect the lipids or acids of interest.

Biological Matrix	Lipid or Acid Detected	Laboratory Method	References
Muscle tissue	Sterols (cholesterol, 5α-cholestanol, and cholestanone) SFA (C18:0 and C16:0) and UFA (C18:1 and C18:2)	GC–MS/MS	[23]
	Choline phosphate	LC-MS	[26]
	PG, PC, PtdE, PlsCh, PlsEtn, and VLCFA	HR-MS	[27]
	Sterols (Cholesterol sulfate and DHEA sulfate), PlsE, PlsCh, PG, and FA (VLCFA and UFA)	Shotgun lipidomic analysis	[35]
Bone	PC	HR-MS	[21]
	LysoPC, PI, and PC	LC–MS	[13]
Textile in contact with decomposing remains	SFA, UFA, and bile acids	GC–MS/MS	[20]
Textile in contact with corpse	SFA (C14:0, C16:0, and C18:0) and UFA (C16:1, C18:1, and C18:2)	GC–MS	[29]
Soil from burial sites	Sterols (coprostanol and epicoprostanol)	ICP-OES-/ICP-MS	[25]
	SFA (C16:0 and C18:0) and UFA (C18:1)	GC-FID	[28]
	Sterols (cholesterol)	GC–MS/MS	[32]
	FA (C14:0, C16:0, C18:0, C18:1, C18:2, and 10-OHC18:0)	GC–MS and GC-FID	[33]
	SFA (C16:0 and C18:0)	GC-FID	[31]
	VFA (C3:0, isobutyric acid, n-butyric acid, isovaleric acid, and n-valeric acid)	GC–MS	[34]
	Sterols (Cholesterol, β-sitosterol, and coprostanol)	GC–MS	[36]
	SFA (C14:0, C15:0, C16:0, C17:0, and C18:0), UFA (C18:1, C18:2, and C16:1), and hydroxy fatty acids (10-OH-C18:0)	LC–MS	[37]
	FA (TG, SFA, UFA, salts of acids, and hydroxy fatty acids)	FTIR-ATR	[38]
	FA (TG, SFA, and UFA)	GC–MS and FTIR	[39]
	SFA (C14:0, C16:0, and C18:0), UFA (C18:1), and hydroxy fatty acids (10-OH-C18:0)	GC–MS	[40]
	SFA (C14:0, C16:0, and C18:0), UFA (C16:1 and C18:1), and hydroxy fatty acids (10-OH-C18:0)	GC–MS	[41]
	SFA (C14:0, C16:0, and C18:0), hydroxy fatty acids, and fatty acid salts	DRIFTS	[24]
Adipose tissue	FA	FTIR-ATR	[30]
Animal blood	SFA (C16:0 and C18:0) and UFA (C18:1)	GC–MS	[14]
Soft tissue	SFA (C18:0 and C16:0) and UFA (C18:1, C18:2, and C16:1)	GC–MS	[2]

FA: fatty acid; UFA: unsaturated fatty acid; SFA: saturated fatty acid; FTIR-ATR: Fourier transform spectrophotometer attenuated total reflectance; C14:0: myristic acid; C15:0: pentadecanoic acid; C16:0: palmitic acid; C16:1: palmitoleic acid; C17:0: margaric acid; C18:0: stearic acid; C18:1: oleic acid; C18:2: linoleic acid; DRIFTS: diffuse reflectance mode infrared spectroscopy; FTIR: Fourier transform spectrophotometer; GC-FID: gas chromatography with flame ionizer detector; GC–MS: gas chromatography/mass spectrometry; GC–MS/MS: gas chromatography with triple quadrupole mass spectrometry; HR-MS: high resolution mass spectrometry; ICP-OES/ICP-MS: inductively coupled plasma optical emission spectrophotometry/inductively coupled plasma mass spectrometry; LC–MS: liquid chromatography/mass spectrometry; LysoPC: Lysophosphatidylcholine; PC: phosphatidylcholine; PG: phosphatidylglycerol; PI: phosphatidylinositol; PlsCh: choline plasmalogen; PlsEtn: ethanolamine plasmalogen; PtdE: phosphatidylethanolamine; TG: triglyceride; VFA: volatile fatty acid; VLCFA: very long chain fatty acid; 10-OH-C18:0: 10-hydroxystearic acid.

**Table 3:** Review of studies on lipid variations and their relationship with PMI. In the case of sex in human samples.

Ref.	Sample	Human/Animal	Cadaver Sample Data						Postmortem Sampling (Days)	Category	Lipid Type	Results
			Sex	PMI of the Samples	Decomposition Environment	Age	Weight (kg)	Cause of Death				
[21]	The medial calcaneus, proximal tibia, and vertebral body (fourth lumbar)	Human	-	Between less than 1 year and 30 years	External environment	-	-	-	Sampling every 6 months for 24 months	GPL	PC (34:1) PC (34:2) PC (36:1) PC (36:2) PC (36:4)	Decrease with PMI between the first and sixth month since the beginning of this study.
[13]	Anterior midshaft tibia	Human	4 W	2, 3, and 10 days	Two subjects in shallow open pits	Between 61 and 91 years old	-	-	Days 0, 219, 790, and 872	GPL	LysoPC PI PC	Drastic reduction in intensity of markers between the fresh state and the state after decomposition.
					Two subjects buried in pits							
[26]	Biceps femoris muscle tissue.	Human	1 W	-	Refrigerated	69	-	Suicide	Days 11, 12, 13, 14, and 15	GPL	Choline phosphate	Shows an increasing pattern with PMI from day 7 to 19 postmortem.
			5 M	-	Refrigerated	60	-	Hemorrhagic stroke	Days 7, 8, 9, and 10			
					Refrigerated	62	-	Pulmonary embolism	Days 11, 12, 13, 14, and 15			
					Refrigerated	69	-	Metastatic nonsmall cell lung cancer	Days 12, 13, 14, 15, and 16			
					External environment	59	-	Acute respiratory distress syndrome	Days 3, 4, and 5			
					External environment	60	-	Cardiovascular disease	Days 18 and 19			
[27]	Vastus lateralis muscle	Human	-	-	External environment	-	-	-	Daily samples up to 2000 ADD or until the muscle is no longer available	GPL	PG (34:0) PC (36:2) PtdE (36:4)	Decrease with PMI. Regression models were developed with PG 34:0 and PtdE 36:4.
										Plasmalogen	PlsCh (34:2) PlsEtn (36:4)	Decrease with PMI.
										VLCFA	-	Increase with PMI.
[35]	Skeletal muscle	Human	-	-	-	-	-	-	Days 1, 9, and 24	Sterol sulfate	Cholesterol sulfate and DHEA sulfate	Increase with PMI.
										Plasmalogen	PlsE (36:1) and (40:6) PlsCh (34:1) and (36:4)	Decrease with PMI.
										GPL	PG	Decrease with PMI.
										5FA	VLCFA	Decrease with PMI.
											PUFA	Increase with PMI.
[26]	Biceps femoris muscle tissue	Animal (rat)	8 M	0 days	Two of them immediately dissected	Adult	-	Euthanasia	Day 0, 1, 2, and 3	GPL	Choline phosphate	There is an increase during the investigation period (3 days).
					Six remaining were dissected for 3 days.							

W: women and M: men, in animal samples. (-) Not indicated. ADD, Accumulated degree days; DHEA sulfate, dehydroepiandrosterone sulfate; FA, fatty acid; GPL, glycerophospholipid; LysoPC, Lysophosphatidylcholine; PC, phosphatidylcholine; PG, phosphatidylglycerol; PI, phosphatidylinositol; PlsCh, choline plasmalogen; PlsE, ethanolamine plasmalogen; PlsEtn, ethanolamine plasmalogen; PMI, postmortem interval; PtdE, phosphatidylethanolamine; PUFA, Polyunsaturated fat; VLCFA, very long-chain fatty acid. “Lipid (N:n)” indicates the total number of carbons that the molecule is composed of, and n is the number of double bonds it has.

**Table 4:** Review of studies on fatty acid variations and their relationship with PMI.

Ref.	Sample	Human/Animal	Cadaver Sample Data						Postmortem Sampling *	Marker Category	Lipid Type	Results
			Sex	Decomposition Environment	Age	Weight	BMI	Cause of Death				
[23]	The tissue of the upper arm, lower abdomen/torso region, and the buttocks/upper thigh (right side)	Human	2 M	External environment (summer). One donor was previously frozen, while the other was not.	68 and 77 years old	104 and 90 kg	32.5 and 30.8 (kg/cm^2^)	-	Days 0, 2, 4, 6, 8, 8, 10, 10, 12, 14, 14, 17, 20, 24, 48, and 69	Sterols	Cholesterol, 5a-cholestanol, and cholestanone	They were not suitable biomarkers for the estimation of PMI since there is no specificity among donors in the long term.
										SFA	C18:0 C16:0	Good biomarkers for PMI estimation.
										MUFA and PUFA	C18:1 C18:2	
[24]	Adipose tissue.	Animal (rat)	462 M	Controlled environment at 5 °C and humidity 50 ± 5%.	-	24–26 g	-	Cervical dislocation	From day 0 to 10 and day 14	FA	-	An increase in free fatty acids and a decrease in fatty acid-glycerol bonds with PMI are detected. Regression models are obtained for the estimation of the PMI.
				Controlled environment at 15 °C and humidity 50 ± 5%.					From day 0 to 10 and day 14			
				Controlled environment at 25 °C and humidity 50 ± 5%.					From day 0 to 10 and day 14			
				Controlled environment at 35 °C and humidity 50 ± 5%.					Days 0, 0.5, 1, 1.5, 2, 2.5, 3, 3.5, and 4			
[30]	Blood.	Animal (rat)	42 M	In plastic bags, in an environment with constant temperature and humidity at 12 ± 2 °C and 50% ± 10%, respectively.	-	-	-	Suffocated	Hour 0, 3, 6, 12, 12, 24, 48, and 72. Minimum one day, maximum two days.	SFA and MUFA	C16:0 C18:0 C18:1	An increase in compounds is determined as the PMI progresses. Regression models are obtained for the estimation of the PMI.
			42 F									
[14]	The soft tissue of the upper and lower torso.	Animal (*Sus scrofa domesticus*)	-	External environment with presence and absence of insects.	Adults	20–30 kg	-	Head bolt	Maximum 111 days	SFA	C18:0 C16:0	An increase in compounds is determined as the PMI progresses.
										MUFA and PUFA	C18:1 C18:2 C16:1	A decrease in compounds is observed as the PMI progresses.
[20]	Textile in contact with decomposing corpse. Control samples are taken.	Human	2 M	One donor is deposited in summer, and the other one in winter.	84 and 86 years old	63 and 100 kg	20.8 and 30.2 (kg/cm^2^)	Alzheimer Metastatic bladder cancer	Days 0 and 105	SFA, UFA, and bile acids	C16:1, C18:2, C16:0, and C18:1, Deoxycholic and lithocholic acids	In summer, a decrease in UFA and an increase in SFA analogs is detected. An increase in bile acids is also detected. In winter, there is a minimal presence of SFAs and UFAs.
[31]	The soil in contact with the corpse (soil in contact with large muscle mass).	Human	M	External environment.	Adult	-	-	-	* ADD for the 90 days prior to the discovery of the corpse are taken.	VFA	C3:0 Isobutyric acid n-butyric acid Isovaleric acid n-valeric acid	These fatty acids are detected in soil samples.
[39]	Soil from grave exhumations (area under the trunk region of the remains).	Human	-	Buried in graves.	-	-	-	-	Graves of different PMI (6 and 12 years)	SFA and MUFA	C14:0 C16:0 C18:0 C18:1 10-OH-C18:0	A lower concentration of fatty acids is detected in 12-year-old samples and higher in 6-year-old samples.
[25]	Soils of burial sites. Abdominal fat is buried with muscle and skin.	Animal (*Sus scrofa*)	-	Burial in a vessel with animal and soil sample (acid pH = 3.74).	-	-	-	-	Days 0, 3, 5, 7, 15, 15, 17, 21, and 28	SFA and MUFA	C16:0 C18:0 C18:1	An increase in the concentration of these FAs is detected in the early stages of decomposition and a decrease towards the end of the test period. Highest concentration of C16:0, followed by C18:0 and C18:1.
				Burial in a vessel with animal and soil sample (slightly alkaline pH = 7.45).								An increase in the concentration of these FAs is detected later in the decomposition. Highest concentration of C18:1, followed by C18:0 and C16:0.
[29]	Textile in contact with decomposing corpse.	Animal (*Sus scrofa*)	-	Burial of the animal with the textile remains in summer.	-	-	-	-	Minimum day three, maximum day 499	SFA and UFA	C14:0 C16:0 C16:1 C18:0 C18:1 C18:2	Increase in the percentage of SFA in the first stages (up to day 31) and then maintain it until the end of the trial.
				Burial of the animal with the textile remains in winter.								There is a decrease in the percentage of SFA at the beginning of the decomposition until day 65; then, there is an increase in the percentage of SFA until the end of the trial.
[33]	Sandy soil	Animal (*Sus scrofa*)	-	Pig fatty flesh is buried in a vial with sandy soil.	-	-	-	-	Days 0, 3, 5, 10, 20, 50, and 90	SFA	C16:0 C18:0	It increases in concentration, reaching a maximum on day 20. Subsequently, it decreases until the end of the trial (day 90).

ADD: accumulated degree day; BMI: body mass index; C14:0: myristic acid; C16:0: palmitic acid; C16:1: palmitoleic acid; C18:0: stearic acid; C18:1: oleic acid; C18:2: linoleic acid; 10-OH-C18:0: 10-hydroxystearic acid; FA: fatty acid; MUFA: monounsaturated fatty acid; PMI: postmortem interval; PUFA: polyunsaturated fatty acid; SFA: saturated fatty acid; UFA: unsaturated fatty acid; VFA: volatile fatty acid. In the case of sex in human samples; in animal samples, M is male, and F is female. (-) Not indicated. * Day 0 is when the samples are deposited, and the test begins.

**Table 5:** Analysis of forensic soil sample studies.

Ref.	Sample	Human/Animal	PMI	Lipid Markers	Type	Results	Use
[22]	Soil of a grave at different depths	Human	Unknown	Sterols	Coprostanol Epicoprostanol	The presence of these markers is detected in the grave samples and is absent in the control samples.	Determination of burial sites.
[28]	Soil in the burial area (under the corpse), at different distances from the grave.	Human	14 days	Sterols	Cholesterol	The presence of cholesterol is detected in the different samples taken from the grave but not in the control samples.	Determination of burial sites.
[32]	Sampling of the soil under the thorax and abdomen of the corpse at different depths and taking control samples.	Human	Estimated 11–18 days. Left in the ground until 358 days later	FA	C14:0, C16:0, C18:0, C18:1, C18:2, and 10-OH-C18:0	With a PMI of 18 days, these FAs are detected, but in slightly higher concentrations in the thorax compared to the abdominal area (5–330 mg/g and 5–52 mg/g). The concentration of these compounds decreases with increasing depth. In upper samples, C16:0 and 10-OH-C18:0 predominate. With a PMI of 358 days, SFA dominates over UFA and 10-OH-C18:0.	Determination of burial sites.
[31]	The soil in contact with the corpse (soil in contact with large muscle mass).	Human	Unknown	VFA	Propionic acid Isobutyric acid N-butyric acid Isovaleric acid N-valeric acid	The presence of these compounds is detected in samples taken from soil under decomposing remains.	Determination of burial sites and estimation of PMI.
[36]	Cemetery soil in areas adjacent to graves with skeletal corpses. Soil samples from burial areas with adipocere.	Human	Unknown	FA	C14:0, C15:0, C16:0, C17:0, C18:0, C18:1, C18:2, C16:1, and 10-OH-C18:0	The presence of the markers was detected in all the samples taken in the cemetery and the adipocere samples.	Determination of burial sites. The presence of 10-OH-C18:0 is detected in soil samples.
[39]	Soils from grave exhumations (area under the trunk region of human remains) and control sampling.	Human	Between 6 and 12 years old	FA	C14:0, C16:0, C18:0, C18:1, and 10-OH-C18:0	These compounds are detected in the exhumation soil samples, not the control samples.	Determination of burial sites with adipocere and estimation of PMI.
[40]	Grave soils with adipocere after exhumation of corpses and forensic burials, with differences in burial depth (1.2–1.8 m) and humid and dry environments.	Human	Exhumations between 5.5 and 50 years. Forensic burials: unknown	FA	C14:0, C16:0, C18:0, C18:1, C16:1, and 10-OH-C18:0	The presence of the markers is detected in the grave samples, but there is a total absence of the markers in the control samples. The 10-OH-C18:0 is only detected in the 13-, 22- and 26-year-old PMI samples and humid environments. Difference in wet and dry environments is observed. Higher C16:0 and lower C18:0 concentrations are detected in soils with humid environments.	Determination of burial sites with adipocere.
[41]	Soils with adipocere of exhumations in cemeteries and control sampling.	Human	Three samples aged 13, 22, and 23 years 27 months	FA	C14:0, C16:0, and C18:0 Hydroxy acids Fatty acid salts	Fatty acids are present in all samples, with the primary fatty acid being C16:0.	Determination of burial sites with adipocere.
[34]	Soils of burial areas (depth of 40 cm) of four pigs.	Animal (*S. s. domesticus*)	3 and 6 months after burial	Sterols	Cholesterol β-sitosterol Coprostanol	There is an increase in the presence of cholesterol and β-sitosterol relative to control samples at three months of burial. Coprostanol can also be detected in some samples, but it is absent in control samples. At six months, a decrease in cholesterol and β-sitosterol concentration is detected.	Determination of burial sites with adipocere.
[37]	Adipocere samples from burial areas of animal adipose tissue. Different environments: silty soil and coffin simulation.	Animal (*S. s. domesticus*)	12 months of burial	FA	Triglycerides Saturated and unsaturated fatty acids Fatty acid salts Hydroxy acids	A difference is detected between the profile obtained by analyzing adipose tissue and adipocytes. In adipose tissue, triglycerides are mainly present. In adipocere samples, triglycerides disappear, and FA, fatty acid salts, and hydroxy acids appear.	Determination of burial sites with adipocere.
[38]	Soil below the decomposition remains. Adipocere samples directly from the cadaver (abdominal area and lower thorax) and control sampling.	Animal (pig)	Different decomposition times (5, 6, 8, 8, 13, and 14 months)	FA	Triglycerides Saturated (C16:0 and C18:0) and unsaturated fatty acids	It can determine the adipocere decomposition profile. Triglycerides are degraded, with decreasing concentration and increasing SFA and UFA. Subsequently, there is a decrease in UFA with an increase in C16:0. This is followed by a further decrease in UFA with a consequent increase in C18:0.	Determination of burial sites and adipocere formation process

C14:0: myristic acid; C15:0: pentadecanoic acid; C16:0: palmitic acid; C16:1: palmitoleic acid; C17:0: margaric acid; C18:0: stearic acid; C18:1: oleic acid; C18:2: linoleic acid; 10-OH-C18:0: 10-hydroxystearic acid; FA: fatty acid; PMI: postmortem interval; SFA: saturated fatty acid; UFA: unsaturated fatty acid; VFA: volatile fatty acid.
